# Effect of Reassuring Information About Musculoskeletal and Mental Health Complaints at the Workplace: A Cluster Randomized Trial of the atWork Intervention

**DOI:** 10.1007/s10926-018-9786-6

**Published:** 2018-05-21

**Authors:** Tone Langjordet Johnsen, Hege Randi Eriksen, Valborg Baste, Aage Indahl, Magnus Odeen, Torill Helene Tveito

**Affiliations:** 1 Division of Physical Medicine and Rehabilitation, Vestfold Hospital Trust, POB 2168, 3103 Tønsberg, Norway; 2 Uni Research Health, POB 7810, 5020 Bergen, Norway; 3 Department of Sport and Physical Activity, Western Norway University of Applied Sciences, Bergen, Norway; 4 Department of Health, Social and Welfare Studies, University College of Southeast Norway, Horten, Norway

**Keywords:** Sick leave, Subjective health complaints, Employee health, Mental health, Back pain, Workplace, Social support, Randomized controlled trial

## Abstract

*Purpose* The purpose of this study was to investigate the possible difference between the Modified atWork intervention (MAW) and the Original atWork intervention (OAW) on sick leave and other health related outcomes. atWork is a group intervention using the workplace as an arena for distribution of evidence-based knowledge about musculoskeletal and mental health complaints. *Methods* A cluster randomized controlled trial with 93 kindergartens, comprising a total of 1011 employees, was conducted. Kindergartens were stratified by county and size and randomly allocated to MAW (45 clusters, 324 respondents) or OAW (48 clusters, 313 respondents). The randomization and intervention allocation processes were concealed. There was no blinding to group allocation. Primary outcome was register data on sick leave at cluster level. Secondary outcomes were health complaints, job satisfaction, social support, coping, and beliefs about musculoskeletal and mental health complaints, measured at the individual level. *Results* The MAW group reduced sick leave by 5.7% during the intervention year, while the OAW group had a 7.5% increase. Overall, the changes were not statistically significant, and no difference was detected between groups, based on 45 and 47 kindergartens. Compared to the OAW group, the MAW group had a smaller reduction for two of the statements concerning faulty beliefs about back pain, but believed less in the hereditary nature of depression. *Conclusions* The MAW did not have a different effect on sick leave at cluster level compared to the OAW. *Trial registration*https://Clinicaltrials.gov/: NCT02396797. Registered March 23th, 2015.

## Introduction

Subjective health complaints (SHC), such as back pain and reports of feeling anxious or depressed, are prevalent in the general population in Norway [1, 2] and the comorbidity between these health complaints are high [3, 4]. Preventing the occurrence of SHC appear to be a difficult undertaking, despite long-term attempts from the healthcare services. These health complaints seem to be a part of human life, and might be impossible to avoid [5–7]. In some cases, SHC may impact a person’s ability to function as usual [2, 8], and musculoskeletal and mental disorders are the two major diagnostic groups reported for sick leave and disability pension in Norway [9, 10]. Accordingly, the economic consequences of musculoskeletal and mental disorders are high, both for society, the workplace, and the person affected [11, 12]. Equally important are also the negative health consequences workplace exclusion may have for the individual.

Back pain is the largest single cause for sick leave in Norway, but in the last decade sick leave due to mild mental disorders have had a rapid increase, and is today one of the major health challenges in the Norwegian society [9, 12]. The duration of sick leave due to mental disorders is generally longer than for musculoskeletal disorders [13], and mental disorders also account for an average of one-third of all disability pensions, with anxiety and depression being the diagnostic groups contributing to most of the lost working years [10].

There is increasing evidence suggesting that work is good for health, and especially for mental health [11, 14, 15]. Accordingly, it is important to develop effective interventions aiming to improve or sustain labor market participation for employees experiencing SHC. Because SHC appear to be a part of human life, there is a need for interventions aiming to influence the perception and management of SHC and not solely focus on interventions aiming to prevent occurrence. There is evidence that workplace interventions directed towards influencing employees’ perceptions of SHC can lead to positive outcomes, such as reductions in sick leave [16, 17].

atWork is a workplace intervention aimed at reducing the negative consequences of SHC [16, 18]. This is done by providing evidence-based knowledge to all employees and managers, aiming to enable both the individual and the workplace to cope with the consequences of such health complaints. atWork is based on a Brief Intervention [19], a non-injury model [20], and a nondirective social support model [21], and has a theoretical foundation from the Cognitive Activation Theory of Stress (CATS) [22]. atWork is a further development of a workplace intervention providing information about back pain, which showed positive changes in health beliefs and a reduction in sick leave [23]. Originally, atWork was established as a new approach to musculoskeletal complaints, and was effective in reducing sick leave and faulty beliefs about back pain in a large randomized controlled trial [16, 24]. Similarly, a newly conducted trial showed increased odds of work participation among employees who received a comparable intervention to atWork, based on the same BI-principles [17]. Because of the high comorbidity between musculoskeletal and mental health complaints, and the increase in sick leave due to mild mental disorders, the intervention has now been modified to also comprise mental health complaints, with a goal to further reduce sick leave and increase positive effects on other health related outcomes.

The current atWork trial was designed as a cluster randomized controlled trial (RCT) to compare the Modified atWork intervention (MAW) to the Original atWork intervention (OAW) in Norwegian private sector kindergarten employees [18]. A cluster randomization was chosen due to the nature of the intervention; the idea behind atWork is to provide the same information for everyone at the workplace, preferably at the same time, and the workplace sessions were held in groups. The primary aim of the present study was to compare the effect of two workplace interventions on sick leave. The secondary aims were to compare the effect of interventions on health complaints, coping, job satisfaction, social support, and beliefs about musculoskeletal and mental health complaints, measured through individual questionnaires.

## Methods

A parallel, cluster randomized controlled trial with two groups was conducted. The study took place in four Norwegian counties, from May 2014 to January 2017. Clusters were private kindergartens, one kindergarten equaling one cluster. A computer-generated randomization list with a 1:1 allocation ratio was used to randomize clusters into the MAW or the OAW. The full protocol for the trial is published elsewhere [18].

### Sample and Procedure

**Fig. 1:**
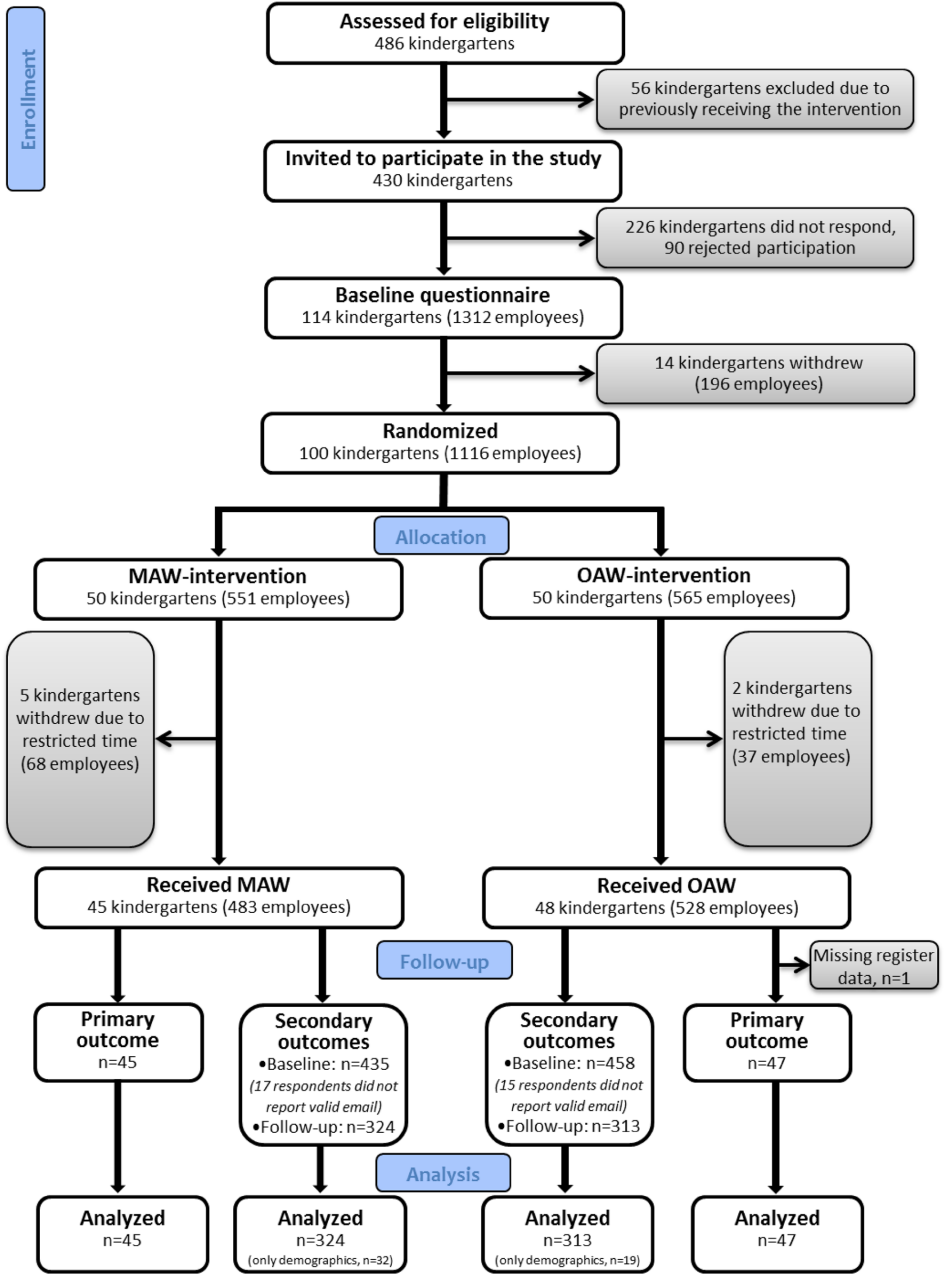
Flowchart of enrollment, allocation, follow-up, and data analysis for the atWork trial, modified from the CONSORT 2010 Statement

A total of 430 private kindergartens in four counties located in Eastern Norway (Telemark, Vestfold, Buskerud, and Akershus) were invited to participate in the study. The enrolment period for the trial was between May 2014 and February 2016. A letter of invitation was emailed to the general manager in the kindergartens, and 114 managers responded that their kindergarten would like to participate. Due to practical reasons, fourteen kindergartens withdrew from the study before randomization. One hundred kindergartens were randomized; 50 kindergartens to the MAW and 50 kindergartens to the OAW (Fig. 1). Seven kindergartens withdrew from the study when it was time to schedule dates for conducting the sessions in the interventions. In six kindergartens the reason for withdrawal was restricted time to participate in the intervention. One kindergarten got a new manager after enrolment, and the new manager wanted time to settle in before participating in a research study. Five of the kindergartens who withdrew from the study had been randomized to MAW and two to OAW, leaving 45 kindergartens in the MAW group and 48 kindergartens in the OAW group (Fig. 1).

Aggregated information on quarterly sick leave for all employees’ per kindergarten, one year before the intervention and the following year, was obtained from the national register in Norway. Register data on sick leave was collected from 92 of the 93 participating kindergartens. One kindergarten was registered as a part of a larger unit, and it was thus not possible to collect sick leave data from only the kindergarten employees. This kindergarten is however included in the questionnaire data analysed, and represents 1.3% of the data material for secondary outcomes.

All employees above 18 years, working at any of the 93 kindergartens agreeing to be a part of the study, totally 1011 employees, were invited to participate in a survey about health and job characteristics. Baseline questionnaires were distributed at enrolment, and questionnaire data was collected using electronically survey software (Qualtrics®) [18]. There were 893 out of the 1011 individual employees who answered the baseline questionnaire. This gives a response rate of 88%. In the baseline questionnaires employees were asked to provide their email address, which was used to distribute follow-up questionnaires. The follow-up questionnaires were distributed to participants 12 months after the kindergarten where they worked had been randomized. Of those responding to baseline questionnaire, 19 employees did not leave an email address and 13 employees left an invalid email address. Follow-up questionnaires were thus distributed to 861 employees, and 637 employees (74%) answered the questionnaire. However, 51 of the respondents only supplied demographic variables. Of the 224 participants not responding, 15 employees reported to the trial coordinator that they did not want to answer the follow-up questionnaire. For the remaining 209 participants, the reason for not responding was unknown. There were more women than men who chose to answer the follow-up questionnaires. They also had higher age and education compared to those who chose not to respond. The distribution of loss to follow up was near equal between intervention groups, and there were no differences in gender, age or education for respondents lost to follow-up.

### Interventions

MAW consisted of (1) one introductory session for managers’ at all organisational levels, health and safety representatives, and local union representatives, (2) two workplace sessions for all employees, one targeting mental health complaints and one targeting musculoskeletal complaints, and (3) one reflection and review session for the participants in the introductory session. OAW consisted of (1) three workplace sessions about musculoskeletal complaints to all employees, and (2) peer support. See study protocol for more details [18]. In the previous atWork trial [16], the peer support function in the OAW was not frequently used and reported to interfere with management roles. Thus, the MAW included two sessions for managers’, health and safety representatives, and local union representatives as an alternative. The reduction from three to one workplace sessions targeting musculoskeletal complaints was based on low attendance rates and participants feedback [16]. The interventions were conducted at group level, and the workplace sessions for all employees were carried out during work hours.

The 93 participating kindergartens received the seminars in the MAW or the OAW between January 2015 and August 2016. Kindergartens did not register for the trial at the same time and the seminars were accordingly carried out in different time periods. The intervention was fully completed by 100% of the kindergartens in the MAW group and 96% of the kindergartens in the OAW group. One kindergarten in the OAW group did not complete the third workplace session and the two peer adviser sessions, and another kindergarten did not attend the second peer adviser sessions because the peer adviser had started on maternity leave. In the MAW group, 93% of the kindergartens had an attendance rate of over 80% for both workplace sessions. In the OAW, 59% of the kindergartens had an attendances rate over 80% for the all three workplace sessions. None of the kindergartens had an attendance rate below 60 for any of the workplace sessions. The kindergarten that did not complete the third workplace sessions had an attendance rate of 78 and 85 percent for the first and second workplace sessions, respectively.

### Primary Outcome Measure, Cluster Level

Primary outcome measure was register data on sick leave for any diagnosis at cluster level (aggregated information on sick leave for employees per kindergarten), collected through the Norwegian Labour and Welfare Association (NAV). The register data comprised quarterly data on the total sum of agreed work days for all employees in each kindergarten and how many of these days were lost due to physician certified sick leave. Agreed work days were the contracted number of days that employees were expected to come to work. Sick leave data were aggregated from all the employees of the participating kindergartens. All register data was collected in June 2017. We did not have ethical approval to collect register data on the seven kindergartens choosing to withdraw from the study.

### Secondary Outcomes, Individual Level

Secondary outcomes were measured at the individual level, through baseline and follow-up questionnaires [18]. *Musculoskeletal complaints* and *pseudoneurological complaints* were measured by two subscales from the Subjective Health Complaints Inventory [25]. The musculoskeletal subscale consisted of eight items (headache, neck pain, upper back pain, low back pain, arm pain, shoulder pain, migraine and leg pain during physical activity) and the pseudoneurology subscale consisted of seven items (extra heartbeats, heat flushes, sleep problems, tiredness, dizziness, anxiety, and sadness/depression). Severity of complaints was rated on a 4-point scale (0—“not at all”, 1—“a little”, 2—“some”, 3—“severe”). The subscales were used as sum scores for the included items. *Low back pain, anxiety*, and *depression* was measured by single items from the same inventory [25], and was dichotomized into no complaints (0 or 1) or substantial complaints (2 or 3).

*Coping expectancies* were measured using the Theoretically Originated Measure of the Cognitive Activation Theory of Stress (TOMCATS) [26]. It consisted of six statements, one representing coping, two representing helplessness and three representing hopelessness, rated on a scale ranging from 1—“completely true” to 5—“not true at all” [26]. All items were reversed so that high scores represent high degrees of coping, helplessness, and hopelessness respectively. To obtain a meaningful comparison to previous research, the questions were recoded from a five to a four point scale, ranging from 1—“not true at all” to 4—“completely true” [16], and mean scores were computed for helplessness and hopelessness.

*Nondirective and directive social support* from co-workers were measured with the Social Support Inventory (SSI) [27, 28]. Seven items measured nondirective social support and three items measured directive social support [29], rated on a scale ranging from 1—“not at all typical” to 5—“very typical”.

*Job satisfaction* was measured using one item from the Global Job Satisfaction scale (GJS) [30], rated on a scale ranging from 1—“very dissatisfied” to 5—“very satisfied”.

*Beliefs about back pain* were measured by seven statements from Deyo’s “back pain myths” [31], presenting untrue and maladaptive beliefs about back pain (listed in Table 4). Statements were rated on a 5-point scale [32], and dichotomized into 0—not believing in the statement (“totally disagree”, ”disagree” and “neither disagrees nor agrees”) or 1—believing in the statement (“agree” and “totally agree”).

*Beliefs about mental health complaints* were measured by 9 statements. The statements were constructed by two of the authors (TLJ and AI), and were based on research and clinical experience related to common worries and beliefs about mental health complaints. Statements are listed in Table 4, and referred to in numerical order below. The first statement was developed to catch embarrassment and stigma being a barrier for openness and help seeking [33]. The second statement aimed at addressing the belief that people don’t recover from mental health complaints [34]. Statement three and four were developed to address the belief that mental health complaints only affect a small part of the population [35]. Statement five and six were developed to address the belief that mental health complaints are purely genetic in nature [36]. Statement seven and eight were developed to address the belief that mental health complaints primarily results from biological pathology and thus best treated with medication [37]. Statement nine aimed at addressing the belief that people experiencing depression are weak [38]. As for beliefs about back pain, all statements were rated on a 5-point scale and dichotomized into 0—not believing in the statement or 1—believing in the statement.

### Sample Size

The sample size estimation was based on a prior atWork trial [16], and we planned to recruit a minimum of 50 units in each intervention group. The calculation for primary outcome, based on the assumptions that changes in sick leave followed a normal distribution, a between group difference of 20% in sick leave (from 9.0 to 7.2%, SD = 3) [39] and a significance level of 0.05, gave 84% power.

### Randomization

The randomization and intervention allocation processes were concealed for the clinicians and researchers and performed at cluster level using a computer generated randomization list stratified by county and size of the kindergarten (small: < 11 employees, large: ≥ 11). The random allocation sequence was generated by the trial statistician. Randomization was performed by the research technician at the randomizing unit (Uni Research Health) after the baseline questionnaire was completed. The trial coordinator then emailed the name, county and size of the kindergarten to the randomization unit and received information about intervention allocation back. The trial coordinator informed the manager of the kindergarten and the personnel performing the intervention about the allocation. Due to the nature of the intervention there was no blinding to group assignment.

### Ethics

The research was carried out in compliance with the Helsinki declaration, and approved by the appropriate ethics committee (Registration 2014/162/REC South East). Informed consent was electronically collected from all participants responding to the study questionnaire.

### Statistical Methods

Descriptive statistics were presented as mean, standard deviation (SD) and percentages. Difference between groups at baseline was tested with Chi-Square tests for gender and education, and independent sample t-tests for age and sick leave. Baseline differences for secondary outcomes were tested with generalized linear models (GLM) with robust variance estimator accounting for clustering of data. Differences on demographic variables between responders and participants lost to follow-up, and also for drop-outs between intervention groups, were tested with Chi-Square testes for gender and education, and independent sample t-test for age.

To analyse the possible different effect of the two interventions on sick leave, a generalized estimating equation (GEE) model with exchangeable correlation structure for kindergarten and robust standard errors was used. The rate of days lost to days agreed for each kindergarten for each quartile was estimated in the model. Total days lost were modelled using a negative binomial distribution to account for overdispersion compared to the simple Poisson model. Log of days agreed were included as offset in the model. Sick leave the year before the interventions was used as baseline; while the 1 year follow up included the quartile the intervention was started. Changes in sick leave between baseline and the intervention year within intervention groups were analysed. Change in sick leave in the MAW group relative to the OAW group was estimated in the model as the interaction between intervention and time. Results from the GEE are presented as incidence rate ratios (IRR) with 95% confidence intervals (CI). As we did not have data to perform an intention-to-treat analysis, only per protocol analyses were performed.

For the continuous secondary outcomes, generalized linear models (GLM) with robust variance estimator to account for clustering of data were used to assess group differences from baseline to follow-up. In the between group analyses, follow-up measures were adjusted for baseline score. For the dichotomous secondary outcomes, a McNemar test was used to test differences between baseline and 1 year after, within intervention groups. Between intervention group difference was tested using multinomial logistic regression with robust variance estimator, to account for kindergarten clusters. All analyses were performed using STATA IC V.14.2 (College Station, Texas, USA).

## Results

Mean age of the respondents were 40.7 years (SD = 10.6), 92.7% were females, and 50.4% had higher education (Table 1). There was no difference in sick leave rates between MAW and OAW at baseline. This was also the case for the majority of secondary outcomes, except for two statements about mental health complaints and the directive social support variable. The MAW group did to a larger degree believe in the hereditary nature of anxiety and depression. The OAW group reported receiving more directive social support from co-workers than the MAW group.

**Table 1:** Demographic characteristics and health status for participants in the two intervention groups, based on baseline questionnaire data

	MAW		OAW		Total	
	n	Mean (SD)	n	Mean (SD)	n	Mean (SD)
Continuous variables						
Age	434	40.4 (10.4)	454	40.9 (10.9)	888	40.7 (10.6)
Musculoskeletal complaints (0–24)	406	4.56 (4.27)	437	4.46 (3.86)	843	4.51 (4.06)
Pseudoneurological complaints (0–21)	406	2.90 (2.96)	436	2.87 (2.80)	842	2.88 (2.87)

	n	%	n	%	n	%
Categorical variables						
Female	435	93.3	458	92.1	893	92.7
Higher education	435	51.7	458	49.1	893	50.4
Substantial low back pain	407	23.8	438	21	845	22.4
Substantial anxiety	406	4.7	436	4.6	842	4.6
Substantial depression	406	4.7	436	6.4	842	5.6

*MAW* modified at Work intervention, *OAW* original at Work intervention

### Primary Outcome

The MAW group had a 5.7% reduction in sick leave during the intervention year, while the OAW group had a 7.5% increase in sick leave compared to baseline. The changes were not statistically significant in either group. There was no difference in sick leave between the groups for the year of the intervention (Table 2).

**Table 2:** Total work days agreed, work days lost and percent sick leave in MAW and OAW, 1 year before the intervention (baseline) and the intervention year

	N	Baseline			1 year			Change within group					Change between groups		
		Days agreed	Days lost	% sick leave	Days agreed	Days lost	% sick leave	%	% points	IRR	95% CI	P-value	IRR	95% CI	P-value
MAW	45	154,028	13,529	8.8	160,160	13,349	8.3	− 5.7	− 0.5	1.06	(0.86–1.28)	0.550	0.97	(0.76–1.24)	0.829
OAW	47	171,488	13,677	8.0	177,056	15,262	8.6	7.5	0.6	1.08	(0.93–1.27)	0.282	1		

The incidence rate ratio (IRR) within group is from the GEE model, accounting for size of kindergartens and dependency in quarterly measurements. IRR between groups is the relative change in sick leave in MAW relative to OAW

*MAW* modified atWork intervention, *OAW* original atWork intervention, *GEE* generalized estimating equation

### Secondary Outcomes

#### Musculoskeletal and Pseudoneurological Complaints

There was no difference in musculoskeletal and pseudoneurological complaints from baseline to follow-up (Table 3). In the MAW group, substantial low back pain was reported by 26.6 and 21% at baseline and follow-up respectively. For substantial anxiety the corresponding numbers were 5.7 and 6%, and for substantial depression 4.4 and 8%. In the OAW group, substantial low back pain was reported by 21 and 18.6% at baseline and follow-up respectively. For substantial anxiety the corresponding numbers were 4.7 and 3.9%, and for substantial depression 6.6 and 5.4%. For substantial low back pain there was a small difference in change between groups (p = 0.043). More of the employees in the MAW group reported being better after the intervention year (16.3% in the MAW, and 10.5% in the OAW), but more of the employees in the MAW group also reported being worse (10.7% in the MAW, and 8.1% in the OAW).

**Table 3:** Mean level of musculoskeletal complaints, pseudoneurological complaints, coping, helplessness, hopelessness, social support and job satisfaction for MAW and OAW at baseline and 1 year after

	MAW				OAW				Between groups
	Baseline		1 year		Baseline		1 year		p-value
	n	Mean (SD)	Mean (SD)	p-value	n	Mean (SD)	Mean (SD)	p-value	
Musculoskeletal complaints (0–24)	252	5.03 (4.32)	4.58 (4.13)	0.055	258	4.29 (3.69)	4.42 (4.12)	0.521	0.254
Pseudoneurological complaints (0–21)	252	3.06 (3.02)	3.03 (3.29)	0.850	258	2.88 (2.79)	2.97 (2.91)	0.600	0.763
Coping (1–4)	253	3.37 (0.40)	3.40 (0.38)	0.202	261	3.36 (0.39)	3.41 (0.39)	0.097	0.741
Helplessness (1–4)	253	1.59 (0.62)	1.66 (0.69)	0.082	259	1.60 (0.68)	1.62 (0.63)	0.598	0.413
Hopelessness (1–4)	253	1.65 (0.53)	1.65 (0.51)	0.915	258	1.62 (0.52)	1.66 (0.52)	0.134	0.460
Nondirective social support (1–5)	266	3.72 (0.76)	3.79 (0.74)	0.064	269	3.76 (0.71)	3.85 (0.69)	**0.037**	0.614
Directive social support (1–5)	265	2.24 (0.70)	2.24 (0.72)	0.945	268	2.36 (0.73)	2.34 (0.73)	0.623	0.397
Job satisfaction (1–5)	276	4.32 (0.64)	4.28 (0.75)	0.342	274	4.36 (0.62)	4.36 (0.65)	0.907	0.382

Test for within and between group differences

P-value < 0.05 when numbers are in bold

*MAW* modified atWork intervention, *OAW* original atWork intervention

#### Coping, Job Satisfaction and Social Support

There were no changes in coping, helplessness, hopelessness, or job satisfaction from baseline to follow-up (Table 3). The OAW group reported receiving more nondirective social support from co-workers after the intervention. There were no differences in change between groups (Table 3).

#### Statements About Back Pain and Mental Health Complaints

For the statements concerning slipped discs and the statement about imagining always identifying the cause of back pain, the reduction in the percentage of employees believing in the statements was smaller in the MAW group compared to the OAW group (Table 4). Both groups had a reduction in employees believing that if you have a slipped disc you must have surgery, that most back pain is caused by injury or heavy lifting, and that everyone with back pain should have a spine radiograph. The OAW group also had a reduction in employees believing that radiographs and newer imaging tests always can identify the cause of pain, and that back pain usually is disabling.

**Table 4:** Percentage of participants agreeing with the statements about back pain and mental health complaints at baseline and 1 year after and test for change in agreement for each statement, for MAW and OAW

	MAW							OAW							Between groups
	Baseline		1 year		Change^a^			Baseline		1 year		Change^a^			p-value
	Total n	Agreed %	Agreed %	p-value	Negative %	Same %	Positive %	Total n	Agreed %	Agreed %	p-value	Negative %	Same %	Positive %	
Statements about back pain															
If you have a slipped disc you must have surgery	284	8.1	3.9	**0.036**	2.8	90.1	7.0	288	13.5	2.4	< **0.001**	0.7	87.5	11.8	**0.038**
Radiographs and newer imaging tests can always identify the cause of pain	284	20.8	15.1	0.056	8.1	78.2	13.7	288	25.4	10.1	< **0.001**	5.2	74.3	20.5	**0.014**
If your back hurts, you should take it easy until the pain goes away	285	6.0	3.9	0.286	2.8	92.3	4.9	289	4.8	2.8	0.238	2.1	93.8	4.2	0.803
Most back pain is caused by injuries or heavy lifting	284	26.8	9.2	< **0.001**	2.5	72.4	25.1	288	34.4	9.0	< **0.001**	1.4	65.2	33.5	0.073
Back pain is usually disabling	283	3.9	1.8	0.180	1.4	95.1	3.5	287	6.6	1.1	< **0.001**	0.7	93.0	6.3	0.209
Everyone with back pain should have a spine radiograph	283	19.8	11.0	**0.001**	5.3	80.6	14.1	288	21.9	8.3	< **0.001**	3.5	79.5	17.0	0.346
Bed rest is the mainstay of therapy	286	0.7	0.7	1.000	3.9	95.4	0.7	288	1.7	0.4	0.219	2.4	95.8	1.7	0.432
Statements about mental health complaints															
Having mental health complaints is embarrassing	288	9.4	8.0	0.557	3.8	91.0	5.2	287	11.2	9.1	0.418	5.6	86.8	7.7	0.286
In most cases, mental health complaints will not pass^R^	288	22.2	15.3	**0.008**	5.6	81.9	12.5	287	25.1	14.6	< **0.001**	5.2	79.1	15.7	0.559
It is uncommon to experience depression^R^	288	8.3	5.9	0.311	4.9	87.9	7.3	287	11.2	5.9	**0.020**	3.8	87.1	9.1	0.580
It is uncommon to experience anxiety^R^	288	17.4	9.7	**0.004**	5.6	81.3	13.2	286	17.5	10.8	**0.007**	4.6	84.3	11.2	0.600
Depression is to a great extent hereditary	287	16.7	13.2	0.223	8.0	80.5	11.5	286	10.1	16.4	**0.010**	10.8	84.6	4.6	**0.001**
Anxiety is to a great extent hereditary	286	12.9	7.3	**0.017**	5.9	83.3	10.8	284	7.8	10.9	0.188	7.7	86.0	6.3	0.113
Depression is best treated with medication	288	3.1	1.7	0.388	7.6	89.6	2.8	287	2.1	1.1	0.508	8.7	89.6	1.7	0.624
Anxiety is best treated with medication	288	1.7	1.7	1.000	8.0	90.3	1.7	285	2.8	0.7	0.070	8.7	88.8	2.5	0.819
Depression is a sign of low willpower	288	3.1	2.1	0.581	1.7	95.5	2.8	286	1.1	3.9	**0.039**	3.5	95.8	0.7	0.094

Percent within intervention group change. Test for difference in change between the intervention groups

P-value < 0.05 when numbers are in bold

*MAW* modified atWork intervention, *OAW* original atWork intervention

^a^Change from baseline to 1 year follow-up; positive change = no longer believing in statement, negative change = started believing in statement

^R^The wording of the statement was reversed from the questionnaire

For the statement claiming that depression to a great extent is hereditary, there was a difference in change between groups. The OAW group had an increase in employees’ believing in this statement, and compared to the OAW group, employees in the MAW group believed less in the hereditary nature of depression (Table 4). Both groups had a reduction in employees believing that people do not recover from mental health complaints and that experiencing anxiety is uncommon. The MAW group also had a reduction in employees believing that anxiety to a great extent is hereditary, while the OAW group had a reduction in employees believing that experiencing depression is uncommon. The OAW group had an increase in employees believing that depression is a sign of low willpower.

## Discussion

### Primary Outcome

The main result of this study was that the MAW did not have a different effect on sick leave compared to the OAW for this sample. There was a small reduction in sick leave in the MAW group and a small increase in sick leave in the OAW group, but overall, the sick leave percentage was relatively stable for both groups during the year before the intervention and the year of the intervention.

The previous atWork trial found a reduction in sick leave when comparing the atWork intervention to a control group not receiving any intervention (treatment as usual) [16]. The same design yielded similar results in a trial investigating the effect of group-based reassuring information about back pain in Danish municipal employees [17]. In the present trial, all participating kindergartens received a version of the atWork intervention, and we did not have data to compare our two intervention groups to a control group not receiving the interventions. Hence, we do not know if the sick leave rates for the kindergartens participating in the trial differ from the sick leave rates of kindergartens treated as usual.

The MAW and the OAW had a theoretical foundation in CATS, and both interventions were aimed at targeting employees’ response outcome expectancies [22]. The interventions also used the same communication model [21], and both targeted back pain. These similarities may make it difficult to detect differences between groups on general sick leave. Sick leave is a multi-causal phenomenon, and successful workplace interventions generally produce small effect sizes [16, 40]. Still, we did not see a systematic decrease in sick leave in either of the intervention groups during the intervention year, as were found in the trial of Odeen et al. [16]. An important difference between these two trials was the study sample, which in the current trial was more homogeneous in regards to gender and occupation. The current trial included only one occupational group, while the previous trial investigated intervention effects among a wide range of occupations. The study from Frederiksen et al. [17] also included employees having different occupations, where the majority of the study sample had manual work tasks. Employees working in the health and social sector, e.g. kindergartens, have higher sick leave rates and higher risk of sick leave compared to other occupations [9, 41]. Thus, it might be that other aspects of the work environment are more important for general sick leave in care occupations, and specific workplace interventions may not produce the same results as in other occupational groups. Compared to the other two trials [16, 17], the current trial also had a higher percentage of female participants, and the rates of sick leave are generally higher for women than for men [9]. The reasons for this difference is debated [42]. Uneven balance in gender distribution at the workplace and difference in social causal explanations for sick leave are suggested explanations [42, 43]. Although the gender gap is poorly understood, there seems to be a consensus that gender plays a role in sick leave and the high percentage of women in this sample may have influenced the results. Furthermore, the sick leave measures were not identical in the mentioned trials. The study of Frederiksen et al. [17] used self-reported days of not attending work, and the study of Odeen et al. [16] included both self-certified and physician certified sick leave. The current trial used physician certified sick leave only.

### Secondary Outcomes

There were few differences between groups on secondary outcomes. However, there were differences in effects on two beliefs about back pain and one belief about depression between intervention groups. Both groups had reductions in employees believing in the back pain myths, indicating that the overall message had been understood and accepted, but for two of the myths there was a smaller reduction in the MAW group compared to the OAW group. This is probably a consequence of difference in time used on back pain in the workplace sessions (1 h in MAW, 3 h in OAW). The difference in back pain beliefs may be relevant for employees’ responses to back pain when it occurs.

Only the MAW group received information and reassurance about mental health complaints, but changes were observed in both groups. For the statement claiming that depression to a great extent is hereditary, there was a difference in change between groups. However, the employees in the MAW group believed more in this statement at baseline than the employees in the OAW group. After the intervention, there was a small decrease in employees believing in this statement in the MAW group, while the OAW group had an increase. Even though this difference in change between groups was statistically significant, it is not likely that the small difference in the percentage of employees agreeing with this statement in the MAW and the OAW would be of practical relevance. The MAW and the OAW both had positive changes in some beliefs about mental health complaints, but the OAW also had some negative changes, moving in the direction of more stigmatizing beliefs. The positive changes in the OAW group may be a consequence of an increased focus on this topic from authorities and the society in general. Also, the general message that SHC are common, generally not harmful conditions, and usual activity may be beneficial, was emphasized in both intervention groups. In the OAW the focus was only on back pain, but the general message may also have affected participants’ beliefs about other SHC.

There was a minor difference in change between the groups for substantial low back pain, where more of the employees in the MAW group reported being either better or worse compared to the OAW group. However, this difference is probably of little practical importance. The OAW group reported receiving more nondirective social support from co-workers after the intervention year. The MAW group also reported receiving slightly more nondirective social support at follow-up, but the change was not statistically significant. The didactic approach used in the interventions was based on a nondirective social support model, demonstrating respect for employees’ autonomy and their capacity to discover and implement solutions to SHC. The subjectivity of these health complaints, and the diversity in experiences and needs, was emphasized in all sessions. Hence, the atWork intervention may facilitate nondirective support of co-workers experiencing SHC.

### Strengths and Limitations

The main strengths of this study were the RCT design, the use of registry data for the primary outcome, the applied setting, and the relatively high response rate. The RCT design provides protection against selection bias and ensures that confounding variables are distributed by chance alone. The use of registry data at cluster level warrants data on all employees in the kindergartens, eliminates loss to follow-up for primary outcome, and bypass the pitfalls of self-report biases [44]. It is a limitation that an intention to treat analysis could not be presented in addition to the per protocol analysis. Several statistical tests was performed, but not adjusted for. The study was performed as a pragmatic trial, evaluating the effect of the interventions under real-life conditions, and the results can thus be generalized and applied to a real-life setting in kindergartens. Ideally, only one new element would have been included in the modified intervention before exploring the effect in a trial. However, this was an evaluation of an intervention under development, and MAW was the model offered to workplaces when the trial was initiated. The response rate for secondary outcomes was relatively high, but baseline differences were found between responders and non-responders to follow up. The characteristics of employees lost to follow up was not different between the intervention groups, reducing the risk of attrition bias [45]. Furthermore, the similarities between the two interventions may have made the trial insufficiently powered to detect differences between groups on general sick leave. A large effort was initiated to recruit more kindergartens to the trial, but unfortunately only 93 kindergartens agreed to participate. Based on completion and participation rates, both the MAW and the OAW are feasible interventions, but the participation rate was generally higher in the MAW compared to the OAW.

## Conclusion

The MAW did not have a different effect on sick leave compared to the OAW in this sample of kindergarten employees. There were few differences also for secondary outcomes, except for some of participants’ belief about SHC. Compared to the OAW group, the MAW group had a smaller reduction for two of the statements concerning faulty beliefs about back pain, but believed less in the hereditary nature of depression. atWork has previously shown positive effects on sick leave and health beliefs, but this study did not provide any indication that the modification of the intervention gave additional positive effects.
